# Adaptability and Life Satisfaction: The Moderating Role of Social Support

**DOI:** 10.3389/fpsyg.2016.01134

**Published:** 2016-07-28

**Authors:** Mi Zhou, Weipeng Lin

**Affiliations:** ^1^School of Psychological and Cognitive Sciences, Peking UniversityBeijing, China; ^2^Department of Human Resource Management, Business School, Nankai UniversityTianjin, China

**Keywords:** individual adaptability, life satisfaction, social support

## Abstract

The purpose of this study was to investigate the moderating role of social support in the relationship between adaptability and life satisfaction. Data were collected from 99 undergraduate freshmen in a Chinese university using a lagged design with a 1-month interval. Results demonstrated that social support moderated the relation between adaptability and life satisfaction, such that the positive relation between adaptability and life satisfaction was stronger for individuals with higher levels of social support than for individuals with lower levels of social support. The theoretical and practical implications of this result are discussed.

## Introduction

The ability to adapt to a rapidly changing environment facilitates positive outcomes (Wilkins et al., 2014). Specifically, adaptability refers to “an individual's ability, skill, disposition, willingness, and/or motivation to change or fit different task, social, or environmental features” (Ployhart and Bliese, 2006). Adaptability is considered to be a key source of mental resources. Individuals with a high level of adaptability can reserve more psychological resources than individuals with a low level of adaptability (Ployhart and Bliese, 2006). Psychological resources are especially important for newcomers who are encountering a totally new environment. The increasingly changing nature of modern life requires individuals to constantly improve their ability to adapt (Ployhart and Bliese, 2006). To adapt to changing circumstances, individuals have to exhibit adaptability both in cognition and behavior (Ployhart and Bliese, 2006).

According to the model of conservation of resources, resources, including both personal and conditional resources, are important factors that can protect individuals from stress in both work and non-work contexts (Hobfoll, 2001). As such, resources would be expected to improve the quality of life. If people attempt to create and maintain personal and conditional resources, they can enhance and avoid losing these resources (Hobfoll, 1989). Accordingly, this is a useful strategy to create pleasure and success.

In the present study, adaptability is considered to be a kind of personal resource that helps individuals to accommodate changing conditions. The environment is often a threat to or can deplete personal resources, but social support can help protect personal resources (Hobfoll, 1989). Therefore, in the present study, social support is considered to be a conditional resource. Thus, based on the construct of conservation of resources, we hypothesize that there is an interaction effect between adaptability and social support on some positive outcomes, such as life satisfaction.

Adaptability and social support are both positive factors in the psychological health domain, and they should jointly promote life satisfaction. However, research to date has failed to examine the interaction effects of these constructs and to investigate the extent to which social support, as a situational factor, strengthens or inhibits the relation between adaptability and life satisfaction. Therefore, the aim of the present study was to determine if social support moderates the relation between adaptability and life satisfaction.

### Adaptability and life satisfaction

Adaptability can be considered a type of self-regulation resource, which is perceived to be a kind of strength that allows control over self (Muraven and Baumeister, 2000, p. 247), and is important in helping individuals adjust to a new environment. Thus, adaptability can promote positive outcomes. For example, there is a positive correlation between adaptability and well-being (Maggiori et al., 2013) and, in university freshmen, adaptation was negatively related to depressive symptoms and level of stress (Dyson and Renk, 2006).

Life satisfaction is an individual's self-evaluation of their quality of life determined using their own rules (Shin and Johnson, 1978). Life satisfaction is the cognitive component of subjective well-being (Pavot and Diener, 1993). Life satisfaction is an important variable and reflects the state of an individual's life as well as their mental state (Pavot and Diener, 1993).

A number of studies have explored the antecedents of life satisfaction (Moksnes and Espnes, 2013; Bastian et al., 2014; Luhmann et al., 2014; Malinauskas et al., 2014; Bourassa et al., 2015; Cheung and Lucas, 2015). From a resource perspective, adaptability can be considered an antecedent of life satisfaction (Wang et al., 2011; Mackey et al., 2013; Stoltz et al., 2013; Martin et al., 2015). A longitudinal study showed that the growth of career adaptability over time was a predictor of the growth of life satisfaction over time (Hirschi, 2009). Santilli et al. (2014) observed a group of adult workers with mild mental disability and found that career adaptability indirectly predicted life satisfaction. Zacher and Griffin (2015) reported that the age of old workers moderated the association between career ability and job satisfaction. Using longitudinal data from high school adolescents, Martin et al. (2013) found that adaptability was positively related to both academic outcomes (i.e., class participation, school enjoyment, and positive academic intentions) and non-academic outcomes (self-esteem, life satisfaction, and sense of meaning and purpose). As such, we hypothesized that, in university freshmen, adaptability would be positively related to life satisfaction.

### The moderating role of social support

Social support refers to information from others expressing concern, love, respect, or value (Kim et al., 2008). Social support has buffering effects that could relieve the pressure felt by individuals (Eaton, 1978). Individuals that received higher levels of social support reported higher levels of well-being (Shakespeare-Finch and Obst, 2011), indicating that social support can result in or promote positive psychological outcomes.

An increasing number of studies are focusing on the effect of social support on life satisfaction (Selda et al., 2013). For example, perceived social support positively affected life satisfaction (Shahyad et al., 2011), school-related social support from parents, peers, and teachers predicted the global life satisfaction of adolescents (Siddall et al., 2013), support from spouses was positively related to life satisfaction (Park and Fritz, 2015), and support predicted life satisfaction, via academic satisfaction (Garriott et al., 2015).

As mentioned above, adaptability and social support are both positive factors in the psychological health domain, and they should jointly promote life satisfaction. However, although research shows that the growth of personal resources (i.e., occupational adaptation) over time can predict the growth of life satisfaction (Hirschi, 2009), limited research has focused on the interactive effects of personal resources (i.e., adaptability) and social resources (i.e., social support) on life satisfaction. Therefore, the aim of the present study was to examine the interaction effects of these constructs to investigate the extent to which social support, as a situational factor, strengthened or inhibited the relation between adaptability and life satisfaction.

Logically, adaptable individuals already have strong ability to adapt to a new environment. If adaptable individuals also receive a high level of social support from the environment, they will have high levels of both internal personal resources and external resources (i.e., social capital). As such, it would be expected that they would be able to adapt well to the environment, and thus have a high level of life satisfaction. By contrast, low levels of social support will weaken the ability of an individual to integrate personal and conditional resources, and thus decrease the level of life satisfaction. In sum, we hypothesize that social support will moderate the relation between adaptability and life satisfaction, such that the positive relation between adaptability and life satisfaction will be stronger for individuals with higher levels of social support.

## Methods

### Participants and procedure

The study was approved by the Institutional Review Board (IRB) of the first author's university. Carefully following the approved protocol, we collected data from 99 undergraduate freshmen in a university in China using a lagged design with a 1-month interval. Freshmen students enrolled in a course were asked to participate in a survey aimed at investigating how they adapted to university life. Participation was voluntary, but the 99 freshmen who participated received extra credit. The survey was confidential.

First-wave data were collected approximately 1 month after university enrollment (time 1) and second-wave data were collected 1 month after the time 1 survey (time 2). Data were collected from 110 individuals at time 1 and 113 individuals at time 2. Ninety-nine individuals completed both surveys; thus the sample size for the analysis was 99. The average age of the 99 participants was 18.25 years (*SD* = 0.85 years, ranging from 15 to 21 years) and 48% were female.

To test the possible bias caused by attrition, we tested whether there were any significant differences in demographic or outcome variables between participants who completed the surveys at both time 1 and time 2 (*n* = 99) and participants who completed the surveys at only time 1 (*n* = 11). There were no significant differences in any variables.

### Measures

Given that all the measures used in the current study were originally published in English, we followed the conventional procedure of translation–back translation (Brislin, 1980) to translate them into Chinese. First, a graduate student fluent in both English and Chinese translated the English scales into Chinese. Then, another graduate student who was also fluent in both English and Chinese re-translated the Chinese versions back into English. Finally, the two English versions of all of the scales were compared with each other to review any inconsistencies. Minor discrepancies between the original and back-translated versions were resolved through discussion between the translators and a professor of psychology.

#### Adaptability

Adaptability was evaluated at time 1 using a 55-item scale developed by Ployhart and Bliese (2006). This scale has been widely used to measure individual differences in adaptability and has been demonstrated to have good reliability and validity in various cultural contexts (e.g., Ployhart and Bliese, 2006; Wessel et al., 2008; Hamtiaux et al., 2013), including China (e.g., Wang et al., 2011). Although, this scale reflects eights facets of adaptability (e.g., learning adaptability, interpersonal adaptability, and adaptability to uncertainty, see Ployhart and Bliese, 2006 for more detail), we treated it as a uni-dimensional measure because the current research focused on how participants' overall adaptability is linked to their life satisfaction. Responses were scored on a seven-point Likert scale ranging from one (*strongly disagree*) to seven (*strongly agree*). Sample items include: “When facing uncertainty, I am able to make effective decisions without all relevant information” and “I believe it is important to be flexible in dealing with new people.” Cronbach's alpha was 0.87.

#### Social support

Social support was evaluated at time 1 using a four-item scale developed by Huang and Lin (2011). This scale is brief and has been shown to have good reliability and validity in Chinese context (Huang and Lin, 2011). Participants were asked to rate the degree to which they had received social support in the past month on a seven-point scale ranging from one (*strongly disagree*) to seven (*strongly agree*). Items include “My friends and I understand each other,” “My friends and I are concerned about each other,” “My friends and I often agree with each other's points of view,” and “My friends and I share emotion with each other.” Cronbach's alpha was 0.88.

#### Life satisfaction

Life satisfaction was evaluated at time 2 using a five-item scale developed by Pavot and Diener (1993). This scale is one of the most commonly used measures of life satisfaction component of subjective well-being, which has been demonstrated to have good reliability and validity in various cultural contexts including China (Pavot and Diener, 1993, 2008). Responses were scored on a seven-point Likert scale ranging from one (*strongly disagree*) to seven (*strongly agree*). Items include “In most ways my life is close to my ideal,” “The conditions of my life are excellent,” “I am satisfied with my life,” “So far I have gotten the important things I want in life,” and “If I could live my life over, I would change almost nothing.” Cronbach's alpha was 0.88.

*Control variable* (rated at time 1): Previous research demonstrated that age and gender are related to life satisfaction (Daig et al., 2009). Therefore, age and gender were included as control variables in our analyses.

## Results

The mean, standard deviation, and reliability of and correlations between variables are presented in Table 1. Adaptability and social support (measured at time 1) were both positively related to life satisfaction (measured at time 2).

**Table 1:** **Means, standard deviations, and correlations between variables (***N*** = 99)**.

	***M***	***SD***	**1**	**2**	**3**	**4**	**5**
1. Gender	1.48	0.50					
2. Age	18.25	0.85	−0.05				
3. Adaptability	4.72	0.68	−0.23^*^	−0.15	(0.87)		
4. Social support	5.50	1.21	−0.25^*^	−0.07	0.30^**^	(0.88)	
5. Life satisfaction	4.37	1.54	−0.03	−0.14	0.39^**^	0.30^**^	(0.88)

*Adaptability and social support were evaluated at time 1. Life satisfaction was evaluated at time 2. Gender was coded as male = 1, female = 2. Alpha reliabilities are provided in parentheses on the diagonal*.

* p < 0.05;

** *p < 0.01*.

Before conducting ordinary least squares regression analyses to test our hypothesis, a number of assumptions must be fulfilled (Keppel and Zedeck, 1989). The relationships between predictors and outcome variable should be linear and the variables normally distributed with equal variances. From visual inspection of the scatterplots, it was concluded that the assumption of linearity was adequately met. Tests for normality demonstrated no violations of assumptions underlying the regressions. We also ran multi-collinearity diagnostics, which indicated that all individual variance inflation factor (VIF) values were below 2 (mean VIF = 1.16), suggesting that multicollinearity did not affect our results.

As such, it is appropriate to use ordinary linear regression to test our hypothesis. Specifically, we used a two-step hierarchical multiple regression analyses. In the first step, control variables (i.e., age and gender), adaptability, and social support were entered into the regression analysis. In the second step, the multiplicative interaction term between adaptability and social support was entered to directly test the current hypothesis about the moderating effect. All predictor variables were centered by subtracting their means. Table 2 shows the results of regression analysis. Adaptability (*b* = 0.74, *SE* = 0.23, β = 0.33, *p* = 0.00) and social support (*b* = 0.29, *SE* = 0.13, β = 0.23, *p* = 0.03) were both positively related to the subsequent measure of life satisfaction. Moreover, the interaction effect between adaptability and social support on life satisfaction was significantly positive (*b* = 0.28, *SE* = 0.13, β = 0.19, *p* = 0.04), indicating that the effect of adaptability on life satisfaction depends on social support. The addition of the interaction effects between adaptability and social support explained additional 4% variance in life satisfaction. *F*-test demonstrated that the addition of this interaction effect was significant (*F* = 4.39, *p* = 0.04).

**Table 2:** **Results of multiple regression (***N*** = 99)**.

	**Life satisfaction (Time 2)**									
	**Step 1**					**Step 2**				
**Variable (Time 1)**	***b***	**SE**	**β**	***t***	***p***	***b***	**SE**	**β**	***t***	***p***
Constant	4.40	0.48		9.13	0.00	4.35	0.48		9.17	0.00
Gender	−0.02	0.31	−0.01	−0.07	0.95	−0.03	0.31	−0.01	−0.10	0.92
Age	−0.15	0.17	−0.08	−0.86	0.39	−0.11	0.17	−0.06	−0.65	0.52
Adaptability	0.72	0.24	0.32	3.05	0.00	0.74	0.23	0.33	3.17	0.00
Social support	0.26	0.13	0.20	1.96	0.05	0.29	0.13	0.23	2.20	0.03
Adaptability × Social support						0.28	0.13	0.19	2.10	0.04
*R*^2^	0.20					0.24				
*Adjusted R*^2^	0.16					0.19				
*ΔR*^2^	0.20					0.04				
F for *ΔR*^2^	5.80					4.39				
*p* for *F*-test	0.00					0.04				

*Gender was coded as male = 1, female = 2. b, unstandardized estimates. SE, standard errors; β, standardized estimates*.

**Figure 1:**
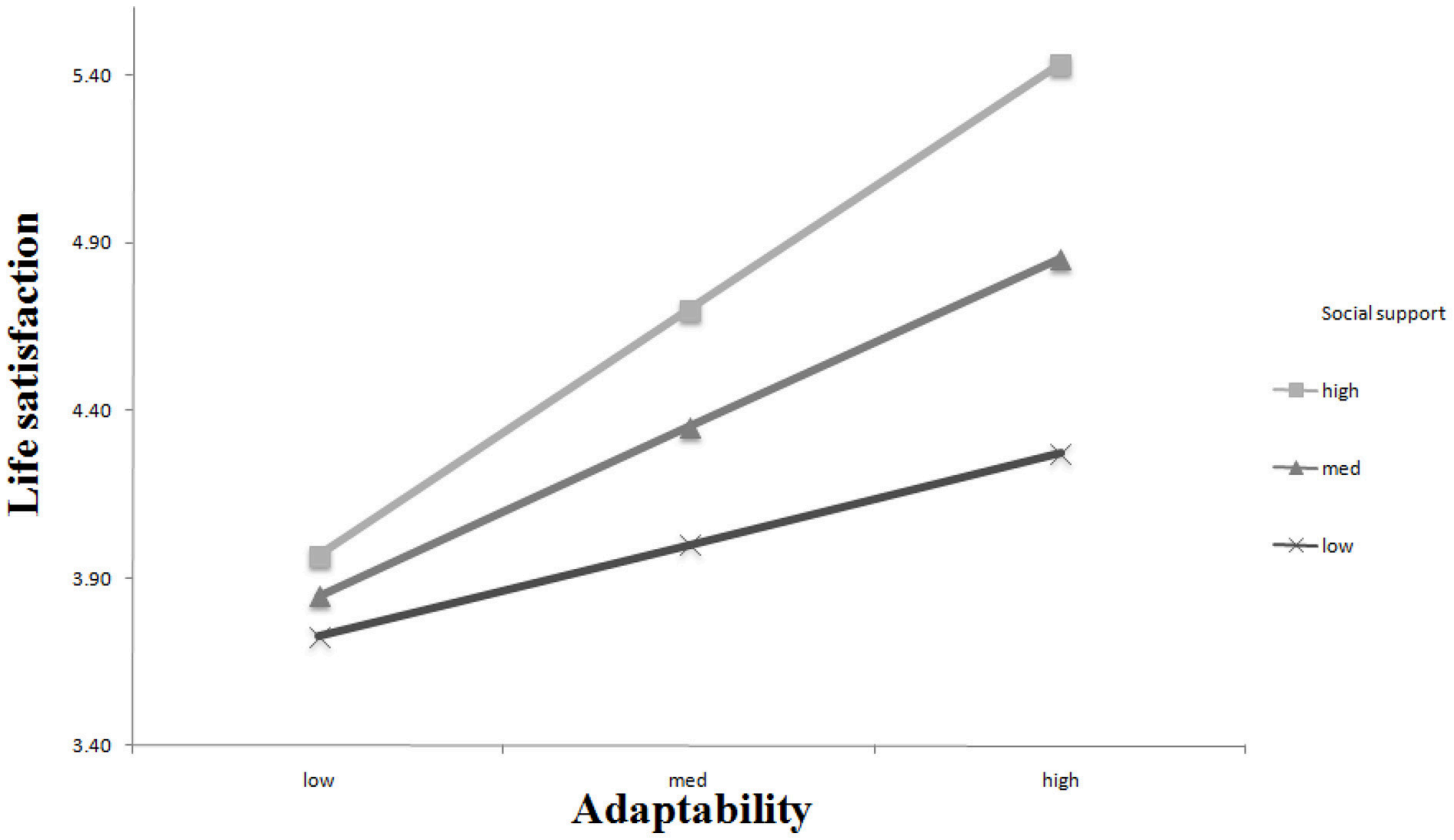
**The moderating effect of social support on the relation between adaptability and life satisfaction**.

We used the online calculator developed by Preacher et al. (2006) to estimate simple slopes, which describe the relations between adaptability and life satisfaction at multiple levels of social support. High, average, and low social support was designated as 1 SD above the mean, mean, and 1 SD below the mean, respectively. A simple slope test demonstrated that the relation between adaptability and life satisfaction was significantly positive when social support was high (*b* = 1.08, β = 0.47, *t* = 3.75, *p* = 0.00) and average (*b* = 0.74, β = 0.33, *t* = 3.08, *p* = 0.00), but was not statistically significant when social support was low (*b* = 0.40, β = 0.18, *t* = 1.44, *p* = 0.15). These results demonstrate that social support strengthened the relationship between adaptability and life satisfaction (see Figure 1), supporting our hypothesis.

## Discussion

In the current study, we explored the interaction effect between adaptability and social support on life satisfaction. Consistent with the conservation of resources theory (Hobfoll, 2001), we found a positive relation between adaptability and life satisfaction. Moreover, we found that social support played a moderating role in the relation between adaptability and life satisfaction. Specifically, the positive relation between adaptability and life satisfaction was stronger for participants who reported higher levels of social support than for participants who reported lower levels of social support.

University freshman are newcomers in the educational setting, and face a new and challenging environment, especially in the first year after enrollment (Park et al., 2012). As such, the adaptation of university freshmen has received a large amount of attention (Park et al., 2012). Adaptability is a type of self-regulation resource and is considered important in helping freshmen to adjust to university life in the first year (Park et al., 2012). Adaptability may influence whether or not an individual can adapt to the new university environment, which is quite different from the high school environment. In university freshmen, adaptation was negatively related to depressive symptoms and level of stress (Dyson and Renk, 2006).

Generally speaking, individuals will take the initiative to adjust their adaptability when facing a new environment or a changing situation. However, in addition to self-adjustment, receiving external support and external resources can also aid quick adaptation to the environment. Social support reflects the level of social resources received from the external environment (Shallcross et al., 2014), and thus can aid quick adaptation to the environment.

The findings of the present study have important implications for positive psychology. Prior studies indicate that adaptability can predict a number of positive outcomes. For instance, Overdale and Gardner (2012) showed that coping adaptability was positively related to performance. Individuals who have stronger adaptability can adjust their own psychological resources to make themselves suitable for the new environment and changing conditions. One previous study showed a direct relationship between adaptability and life satisfaction (Martin et al., 2013), and the present study extends these findings by showing that social support strengthened this relation. Our results suggest that individuals with high levels of both adaptability and social support will be better able to adapt to new and changing social environments, and will thus experience higher levels of life satisfaction.

The results of this study suggest that individuals not only need to improve their adaptability to fit in to a new environment, but also need to strive for social support from others when encountering a new environment or changing situation. Social support will enable them to adapt to the environment better, and a combination of adaptability and social support will have a more positive impact on life satisfaction. Adaptability from within and social support from others are both important to happiness. Modern society has a rapid rhythm and changes quickly. If individuals want to feel satisfied in this modern society, they must adapt to changing situations as soon as possible and receive social support from others. These two kinds of psychology embody the positive attitude that is required to respond to modern society, and will maximize the positive impact of modern society.

## Limitations and direction for future research

This study has several limitations. First, Our research sample was a group of university students that did not include working people. More heterogeneous samples are needed to test the generalizability of our findings. Second, our data were collected from the same source, which might result in common method variance. However, we collected the data using a lagged design. Specifically, the antecedent and outcome variables were measured separately with a 1-month interval in between measurements, and the order of measurements was consistent with the temporal order of variables in our model. This lagged design might mitigate the common method variance (Podsakoff et al., 2003). Moreover, common method variance is unlikely to result in statistical interactions (see Siemsen et al., 2010; Lin et al., 2013), which are the main focus of this study. Thus, we believe that our findings cannot be an artifact of common method variance. Third, the research design of our study would make it difficult to interpret causal inference. Although, we measured the antecedent and outcome variables separately with a 1-month time lag, we cannot directly test the causal direction between our studied variables because we did not measure all variables repeatedly, which is the basic requirement for longitudinal research (Ployhart and Vandenberg, 2010). Thus, future research should use a longitudinal design to provide a stronger inference of the causal relation between adaptability and life satisfaction.

Future research on adaptability could focus on the effects and characteristics of different sub-dimensions of adaptability, examining different factors that influence each sub-dimension. Future research could also examine the developmental trajectory of adaptability. Adaptability may change with the changing environment. It might be interesting to see how individual adaptability develops, as well as how such development affects adaptation outcomes. In addition, future research could further examine the impact of adaptability on other aspects of individual well-being, such as life purpose (Damon et al., 2003; Han, 2015). Life purpose refers to “a stable and generalized intention to accomplish something that is at once meaningful to the self and of consequence to the world beyond the self” (Damon et al., 2003, p. 121). Similar to general life satisfaction that we examined in the current study, the development of life purpose is also closely associated with adaptability and social support (Bundick, 2011; Bundick and Tirri, 2014; Han, 2015). It is possible that adaptability and social support would interactively impacts life purpose. As such, future research could test the generalizability of the interaction effect between adaptability and social support using other well-being outcomes.

## Author contributions

MZ is the first author, who presented the idea and wrote the main part of the manuscript; WL is the corresponding author, who built the structure of the article and is in charge of the whole project.

## Funding

This work is supported by National Natural Science Foundation of China (71502086 and 71532005).

### Conflict of interest statement

The authors declare that the research was conducted in the absence of any commercial or financial relationships that could be construed as a potential conflict of interest.

## References

[B1] Bastian B. Kuppens P. Roover K. D. Diener E. (2014). Is valuing positive emotion associated with life satisfaction? Emotion 14, 639–645. 10.1037/a0036466 24749643

[B2] Bourassa K. J. Sbarra D. A. Whisman M. A. (2015). Women in very low quality marriages gain life satisfaction following divorce. J. Family Psychol. 29, 490–499. 10.1037/fam0000075 25868007 PMC12063491

[B3] Brislin R. W. (1980). Translation and content analysis of oral and written materials, in Handbook of Cross-Cultural Psychology: Methodology, eds Triandis H. C. Lonner W. (Boston, MA: Allyn and Bacon), 389–444.

[B4] Bundick M. J. (2011). The benefits of reflecting on and discussing purpose in life in emerging adulthood. New Dir. Youth Dev. 132, 89–103. 10.1002/yd.430 22275281

[B5] Bundick M. J. Tirri K. (2014). Student perceptions of teacher support and competencies for fostering youth purpose and positive youth development: perspectives from two countries. Appl. Dev. Sci. 18, 148–162. 10.1080/10888691.2014.924357

[B6] Cheung F. Lucas R. E. (2015). When does money matter most? Examining the association between income and life satisfaction over the life course. Psychol. Aging 30, 120–135. 10.1037/a0038682 25621741 PMC4686135

[B7] Daig I. Herschbach P. Lehmann A. Knoll N. Decker O. (2009). Gender and age differences in domain-specific life satisfaction and the impact of depressive and anxiety symptoms: a general population survey from Germany. Qual. Life Res. 18, 669–678. 10.1007/s11136-009-9481-3 19430928

[B8] Damon W. Menon J. Bronk K. C. (2003). The development of purpose during adolescence. Appl. Dev. Sci. 7, 119–128. 10.1207/S1532480XADS0703_2

[B9] Dyson R. Renk K. (2006). Freshmen adaptation to university life: depressive symptoms, stress, and coping. J. Clin. Psychol. 62, 1231–1244. 10.1002/jclp.20295 16810671

[B10] Eaton N. W. (1978). Life events, social supports, and psychiatric symptoms: a reanalysis of the New Haven data. J. Health Soc. Behav. 19, 230–234. 10.2307/2136537 681736

[B11] Garriott P. O. Hudyma A. Keene C. Santiago D. (2015). Social cognitive predictors of first- and non-first-generation college students' academic and life satisfaction. J. Couns. Psychol. 62, 253–263. 10.1037/cou0000066 25730170

[B12] Hamtiaux A. Houssemand C. Vrignaud P. (2013). Individual and career adaptability: comparing models and measures. J. Vocat. Behav. 83, 130–141. 10.1016/j.jvb.2013.03.006

[B13] Han H. (2015). Purpose as a moral virtue for flourishing. J. Moral Educ. 44, 291–309. 10.1080/03057240.2015.1040383

[B14] Hirschi A. (2009). Career adaptability development in adolescence: multiple predictors and effect on sense of power and life satisfaction. J. Vocat. Behav. 74, 145–155. 10.1016/j.jvb.2009.01.002

[B15] Hobfoll S. E. (1989). Conservation of resources: a new attempt at conceptualizing stress. Am. Psychol. 44, 513–524. 10.1037/0003-066X.44.3.513 2648906

[B16] Hobfoll S. E. (2001). The influence of culture, community, and the nested self in the stress process: advancing conservation of resources theory. Appl. Psychol. 50, 337–421. 10.1111/1464-0597.00062

[B17] Huang J.-W. Lin C.-P. (2011). To stick or not to stick: the social response theory in the development of continuance intention from organizational cross-level perspective. Comput. Human Behav. 27, 1963–1973. 10.1016/j.chb.2011.05.00326751769

[B18] Keppel G. Zedeck S. (1989). Data Analysis for Research Designs. New York, NY: Freeman and Company.

[B19] Kim H. S. Sherman D. K. Taylor S. E. (2008). Culture and social support. Am. Psychol. 63, 518–526. 10.1037/0003-066X 18793039

[B20] Lin W. Wang L. Chen S. (2013). Abusive supervision and employee well-being: the moderating effect of power distance orientation. Appl. Psychol. 62, 308–329. 10.1111/j.1464-0597.2012.00520.x

[B21] Luhmann M. Weiss P. Hosoya G. Eid M. (2014). Honey, I got fired! A longitudinal dyadic analysis of the effect of unemployment on life satisfaction in couples. J. Person. Soc. Psychol. 107, 163–180. 10.1037/a0036394 24956318

[B22] Mackey J. D. Ellen B. P. III. Hochwarter W. A. Ferris G. R. (2013). Subordinate social adaptability and the consequences of abusive supervision perceptions in two samples. Leadersh. Q. 24, 732–746. 10.1016/j.leaqua.2013.07.003

[B23] Maggiori C. Johnston C. S. Krings F. Massoudi K. Rossier J. (2013). The role of career adaptability and work conditions on general and professional well-being. J. Vocat. Behav. 83, 437–449. 10.1016/j.jvb.2013.07.001

[B24] Malinauskas R. Dumciene A. Lapeniene D. (2014). Social skills and life satisfaction of lithuanian first- and senior- year university students. Soc. Behav. Pers. 42, 285–294. 10.2224/sbp.2014.42.2.285

[B25] Martin A. J. Nejad H. Colmar S. Liem G. A. D. Collie R. J. (2015). The role of adaptability in promoting control and reducing failure dynamics: a mediation model. Learn. Individ. Differ. 38, 36–43. 10.1016/j.lindif.2015.02.004

[B26] Martin A. J. Nejad H. G. Colmar S. Liem G. A. D. (2013). Adaptability: how students' responses to uncertainty and novelty predict their academic and non-academic outcomes. J. Educ. Psychol. 105, 728–746. 10.1037/a0032794

[B27] Moksnes U. K. Espnes G. A. (2013). Self-esteem and life satisfaction in adolescents-gender and age as potential moderators. Qual. Life Res. 22, 2921–2928. 10.1007/s11136-013-0427-4 23661225

[B28] Muraven M. Baumeister R. F. (2000). Self-regulation and depletion of limited resources: does self-control resemble a muscle? Psychol. Bull. 126, 247–259. 10.1037/0033-2909.126.2.247 10748642

[B29] Overdale S. Gardner D. (2012). Social support and coping adaptability in initial military training. Military Psychol. 24, 312–330. 10.1080/08995605.2012.678243

[B30] Park C. L. Edmondson D. Lee J. (2012). Development of self-regulation abilities as predictors of psychological adjustment across the first year of college. J. Adult Dev. 19, 40–49. 10.1007/s10804-011-9133-z

[B31] Park Y. Fritz C. (2015). Spousal recovery support, recovery experiences, and life satisfaction crossover among dual-earner couples. J. Appl. Psychol. 100, 557–566. 10.1037/a0037894 25222524

[B32] Pavot W. Diener E. (1993). Review of the satisfaction with life scale. Psychol. Assess. 5, 164–172. 10.1037/1040-3590.5.2.164

[B33] Pavot W. Diener E. (2008). The Satisfaction With Life Scale and the emerging construct of life satisfaction. J. Posit. Psychol. 3, 137–152. 10.1080/17439760701756946

[B34] Ployhart R. E. Bliese P. D. (2006). Individual adaptability (I-ADAPT) theory: conceptualizing the antecedents, consequences, and measurement of individual differences in adaptability, in Understanding Adaptability: A Prerequisite for Effective Performance Within Complex Environments, Vol. 6, eds Burke C. S. Pierce L. G. Salas E. (St. Louis, MO: Elsevier Science), 3–39.

[B35] Ployhart R. E. Vandenberg R. J. (2010). Longitudinal research: the theory, design, and analysis of change. J. Manage. 36, 94–120. 10.1177/0149206309352110

[B36] Podsakoff P. M. MacKenzie S. B. Lee J. Y. Podsakoff N. P. (2003). Common method biases in behavioral research: a critical review of the literature and recommended remedies. J. Appl. Psychol. 88, 879–903. 10.1037/0021-9010.88.5.879 14516251

[B37] Preacher K. J. Curran P. J. Bauer D. J. (2006). Computational tools for probing interactions in multiple linear regression, multilevel modeling, and latent curve analysis. J. Educ. Behav. Statist. 31, 437–448. 10.3102/10769986031004437

[B38] Santilli S. Nota L. Ginevra M. C. Soresi S. (2014). Career adaptability, hope and life satisfaction in workers with intellectual disability. J. Vocat. Behav. 85, 67–74. 10.1016/j.jvb.2014.02.011

[B39] Selda K. Ömer Faruk S. Astrid S. Arun T. (2013). Differences in how trait emotional intelligence predicts life satisfaction: the role of affect balance versus social support in India and Germany. J. Happiness Stud. 14, 51–66. 10.1007/s10902-011-9315-1

[B40] Shahyad S. Besharat M. A. Asadi M. Alipour A. S. Miri M. (2011). The relation of attachment and perceived social support with life satisfaction: structural equation model. Proc. Soc. Behav. Sci. 15, 952–956. 10.1016/j.sbspro.2011.03.219

[B41] Shakespeare-Finch J. Obst P. L. (2011). The Development of the 2-way social support scale: a measure of giving and receiving emotional and instrumental support. J. Pers. Assess. 93, 483–490. 10.1080/00223891.2011.594124 21859288

[B42] Shallcross S. L. Frazier P. A. Anders S. L. (2014). Social resources mediate the relations between attachment dimensions and distress following potentially traumatic events. J. Couns. Psychol. 61, 352–362. 10.1037/a0036583 25019539

[B43] Shin D. C. Johnson D. M. (1978). Avowed happiness as an overall assessment of the quality of life. Soc. Indic. Res. 5, 475–492. 10.1007/BF00352944

[B44] Siddall J. Huebner E. S. Jiang X. (2013). A prospective study of differential sources of school-related social support and adolescent global life satisfaction. Am. J. Orthopsych. 83, 107–114. 10.1111/ajop.12006 23330628

[B45] Siemsen E. Roth A. Oliveira P. (2010). Common method bias in regression models with linear, quadratic, and interaction effects. Organiz. Res. Methods 13, 456–476. 10.1177/1094428109351241

[B46] Stoltz K. B. Wolff L. A. Monroe A. E. Mazahreh L. G. Farris H. R. (2013). Adaptability in the work life task: lifestyle, stress coping, and protean/boundaryless career attitudes. J. Indiv. Psychol. 69, 66–83.

[B47] Wang M. Zhan Y. J. Mccune E. Truxillo D. (2011). Understanding newcomers' adaptability and work-ralated outcomes: testing the mediating roles of perceived P-E fit variables. Pers. Psychol. 64, 163–189. 10.1111/j.1744-6570.2010.01205.x

[B48] Wessel J. L. Ryan A. M. Oswald F. L. (2008). The relationship between objective and perceived fit with academic major, adaptability, and major-related outcomes. J. Vocat. Behav. 72, 363–376. 10.1016/j.jvb.2007.11.003

[B49] Wilkins K. G. Santilli S. Ferrari L. Nota L. Tracey T. J. G. Soresi S. (2014). The relationship among positive emotional dispositions, career adaptability, and satisfaction in Italian high school students. J. Vocat. Behav. 85, 329–338. 10.1016/j.jvb.2014.08.004

[B50] Zacher H. Griffin B. (2015). Older workers' age as a moderator of the relationship between career adaptability and job satisfaction. Work Aging Retire. 1, 227–236. 10.1093/workar/wau009

